# Using two on-going HIV studies to obtain clinical data from before, during and after pregnancy for HIV-positive women

**DOI:** 10.1186/1471-2288-12-110

**Published:** 2012-07-28

**Authors:** Susie E Huntington, Loveleen K Bansi, Claire Thorne, Jane Anderson, Marie-Louise Newell, Graham P Taylor, Deenan Pillay, Teresa Hill, Pat A Tookey, Caroline A Sabin

**Affiliations:** 1 Research Department of Infection & Population Health, University College London, Royal Free Campus, Rowland Hill Street, London, NW3 2PF, UK; 2 MRC Centre of Epidemiology for Child Health, UCL Institute of Child Health, University College London, London, UK; 3 Homerton University Hospital NHS Foundation Trust, London, UK; 4 Africa Centre for Health and Population Studies, University of KwaZulu-Natal, Durban, South Africa; 5 Faculty of Medicine, Imperial College, London, UK; 6 Health Protection Agency, Centre for Infections, London, UK

**Keywords:** Data linkage, HIV, Pregnant women, Antiretroviral therapy, Cohort analysis, United Kingdom

## Abstract

**Background:**

The UK Collaborative HIV Cohort (UK CHIC) is an observational study that collates data on HIV-positive adults accessing HIV clinical care at (currently) 13 large clinics in the UK but does not collect pregnancy specific data. The National Study of HIV in Pregnancy and Childhood (NSHPC) collates data on HIV-positive women receiving antenatal care from every maternity unit in the UK and Ireland. Both studies collate pseudonymised data and neither dataset contains unique patient identifiers. A methodology was developed to find and match records for women reported to both studies thereby obtaining clinical and treatment data on pregnant HIV-positive women not available from either dataset alone.

**Results:**

Women in UK CHIC receiving HIV-clinical care in 1996–2009, were found in the NSHPC dataset by initially ‘linking’ records with identical date-of-birth, linked records were then accepted as a genuine ‘match’, if they had further matching fields including CD4 test date. In total, 2063 women were found in both datasets, representing 23.1% of HIV-positive women with a pregnancy in the UK (n = 8932). Clinical data was available in UK CHIC following most pregnancies (92.0%, 2471/2685 pregnancies starting before 2009). There was bias towards matching women with repeat pregnancies (35.9% (741/2063) of women found in both datasets had a repeat pregnancy compared to 21.9% (1502/6869) of women in NSHPC only) and matching women HIV diagnosed before their first reported pregnancy (54.8% (1131/2063) compared to 47.7% (3278/6869), respectively).

**Conclusions:**

Through the use of demographic data and clinical dates, records from two independent studies were successfully matched, providing data not available from either study alone.

## Background

Antiretroviral therapy (ART) used during pregnancy in combination with appropriate management of delivery and avoidance of breastfeeding is highly effective at reducing the risk of mother-to-child-transmission (MTCT) of HIV [1,2]. As a result of this, and an increased life expectancy of those living with HIV [3,4], many HIV-positive women choose to have children [5]. Some do not yet require ART for their own health and use combination ART, or zidovudine monotherapy, for a period during pregnancy to prevent MTCT, repeating short-term ART use in further pregnancies if they still do not need treatment for their own health [6]. Women on ART at conception are recommended to continue treatment throughout pregnancy and after [6]. The implications of exposure to short-term antenatal ART with respect to women’s longer term health and future treatment responses are incompletely understood [7-10].

Adult HIV cohorts have contributed to understanding HIV disease progression and its management, but may not collect data on childbearing or pregnancy status, whilst MTCT cohorts initiate follow-up during pregnancy and rarely collect data on maternal disease progression and treatment post-delivery. In the UK, comprehensive national surveillance of HIV-positive pregnant women is carried out by the National Study of HIV in Pregnancy and Childhood (NSHPC), but data are limited to information available throughout pregnancy and shortly after [11,12]. The UK Collaborative HIV Cohort (UK CHIC) study collates extensive data, recorded as part of a patient’s clinical record, on adults seen for HIV-related care at large HIV clinics in the UK [13]. This provides information on patients’ long-term follow-up, but pregnancy-specific data are not recorded.

In order to study the long-term impact of antenatal ART use on the health of HIV-positive women, collaboration between NSHPC and UK CHIC was established and a methodology developed to find and match records for women reported to both. This paper describes the matching strategy and estimates the completeness of matching and the extent to which HIV-positive pregnant women in UK CHIC were representative of HIV-positive pregnant women in the UK.

## Methods

The NSHPC and UK CHIC datasets were compared to find and match the records of women reported to both i.e. women in UK CHIC who had been pregnant. Initial attempts using only demographic variables (date-of-birth (DOB), country-of-birth (COB), and ethnicity) led to incomplete matching and created false matches; 1575 women were matched, 156 (9.9%) matching multiple records. Therefore, deterministic decision criteria based on both demographic and clinical fields were devised.

### Data collection

The NSHPC surveillance programme collects data on HIV-positive pregnant women from all maternity units in the UK and Ireland (~240 units) under the auspices of the Royal College of Obstetricians and Gynaecologists. A designated individual from each site, typically a midwife or physician, completes standard reporting forms each quarter which are collated at the Institute of Child Health and transcribed into an electronic database. Data collected include: DOB, probable route of infection, ethnicity, COB, date of UK arrival (if born abroad), date of UK HIV-diagnosis, expected and actual dates of delivery, ART use during pregnancy including start and stop dates, pregnancy outcome and first and last CD4 count and viral load assessments during pregnancy. Soundex, a non-unique code derived from the patient’s surname, has been requested since 2008 [14], and is not yet comprehensively provided (3.4%, (306/8932) records included soundex). Further details about NSHPC are available elsewhere [1,15].

The UK CHIC study is an observational cohort of HIV-positive adults (aged 16 and older) attending for clinical care at (currently) 13 large UK clinics (see acknowledgements). Each year electronic data are extracted from patients’ clinical records and transferred securely to the coordinating centre where duplicate records for the same individual, seen at different sites, are merged [13]. Data collected include: DOB, soundex, probable route of infection, ethnicity, COB, date of HIV-diagnosis in the UK, date and result of all CD4 counts and viral load assessments, use of ART including start and stop dates. Further details are available elsewhere [13,16,17].

Initial matching was undertaken in 2009 [18] and repeated in 2010 using updated datasets [19]. The matching process was formalised in 2011, the results of which are presented here. The UK CHIC dataset comprised 8286 women, aged 16–49, seen since 1^st^ January 1996 to 31^st^ December 2009. A restricted NSHPC dataset comprised 8932 women with 11,771 pregnancies starting after 1995 and reported by the end of 2010.

### Dataset linkage using DOB

**Figure 1:**
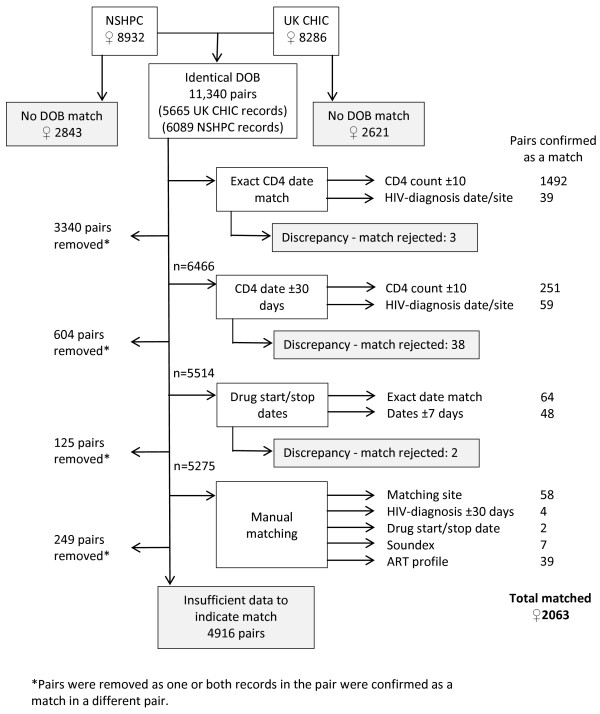
**Criteria used to match records for women reported to NSHPC and UK CHIC.** *Pairs were removed as one or both records in the pair were confirmed as a match in a different pair.

Initially, records in NSHPC with a DOB identical to a record in UK CHIC were ‘linked’ and included in a temporary dataset referred to as the ‘linked DOB dataset’. Some women appeared multiple times in this dataset, as they were linked to multiple records with the same DOB. A series of criteria were then used (as described below) to assess which pairs were a genuine match. If records were confirmed as a ‘match’ (i.e. the NSHPC record referred to the same woman as the UK CHIC record) they were merged and moved to a ‘combined dataset’. All remaining occurrences of these women (i.e. as part of other linked pairs) were removed from the linked DOB dataset. The next stage of matching was then undertaken for pairs of records remaining in the linked DOB dataset (Figure 1). If at any stage a record was matched to multiple records, these were reviewed to identify the strongest match.

### Criteria used to find matching records

#### CD4 date

Initially CD4 date was used to assess whether linked records were a genuine match (Figure 1). Firstly, records with any exactly matching CD4 date and CD4 count within 10 cells/mm^3^ on that date (to take account of rounding up or down to the nearest 10 cells/mm^3^), in their NSHPC and UK CHIC records, were considered a match and moved to the combined dataset. Next, any records with an exactly matching CD4 date but with a CD4 count (on that date) that differed by >10 cells/mm^3^ were considered a genuine match if they had either identical HIV-diagnosis date or were seen for routine HIV care and antenatal care at the same site. Where the difference between CD4 counts was >100 cells/mm^3^ these were manually checked. Sites providing HIV services located close to sites providing maternity care were considered as the same site.

The same criteria were then used to confirm matches among the remaining records in the linked DOB dataset which had CD4 dates that did not match exactly but which were <30 days apart (Figure 1).

#### ART start and stop dates

ART drug start and stop dates were then used to identify further matches from the linked DOB dataset (records not confirmed as a match using CD4 data). Five criteria were used in the following order: where drug and start and stop dates all exactly matched; where drug matched and either the start or stop date exactly matched; where drug did not match or was missing but start and stop dates exactly matched; where either start or stop date exactly matched and records were reported from the same site; and where either start or stop date exactly matched and HIV diagnosis date matched. The process was repeated for records without exactly matching ART start and stop dates but where the respective dates were ≤7 days apart (Figure 1).

#### Manual checking

Finally, records remaining in the linked DOB dataset (records not confirmed as a match using CD4 or ART data) were manually checked to find further matches, using fields including: COB, ethnicity, HIV-diagnosis date, viral load and date, ART start and stop dates and site of care. Records were selected for manual checking if they had been reported from the same site, had HIV-diagnosis dates ≤30 days apart, any drug start or stop date match, matching soundex or an ART profile, in UK CHIC, which indicated they may have had a pregnancy. This included women with an ART start date in UK CHIC during the pregnancy (after the first trimester) and who either started zidovudine monotherapy or combination ART with CD4 > 350 cells/mm^3^ or who had “pregnant” reported as a reason for starting or stopping ART (in UK CHIC).

#### Discrepancy checking

At each stage, before records were merged and moved to the combined dataset, matched records with a discrepancy in COB or ethnicity, variables collected by both studies, were manually checked. Records were also checked if there was a date of death (in UK CHIC) before the estimated date of conception, if women were reported as drug naïve in UK CHIC after antenatal ART start dates in NSHPC or had a date of UK arrival (in NSHPC) after a CD4 count or viral load assessment in UK CHIC. Records with a discrepancy were kept as a match (and assumed to be due to typographic error) if they had sufficient data in agreement in other fields, such as viral load and HIV-diagnosis date, to indicate that they were a genuine match. Discrepancy checking resulted in 43 matched pairs being un-matched.

Where there was a discrepancy in fields collected by both studies, records were examined to identify which data should be used, as described below.

#### HIV-diagnosis date

The earliest HIV-diagnosis date from either study was used unless one date was either 1^st^ January or 30^th^ June (proxy dates used when only year of diagnosis is known/reported) and the later date was during the same year, in which case the later date was used (n = 116). The earliest CD4 count, viral load assessment or ART start date was used if no HIV-diagnosis date was available (n = 4), or preceded the earliest HIV-diagnosis date (n = 78).

#### Region of birth

Region of birth (ROB) was categorized using COB, as defined by the World Health Organization [20]. Records with discrepant ROB (n = 98) were categorized as the non-European region if region was European in one dataset and non-European in the other (n = 96) (94 of which had UK as COB in one study), otherwise ROB was categorized as ‘Not Known’ (n = 2).

### Ethnicity

Where ethnicity was somewhat discrepant, for example ‘black-other’ versus ‘other’ (n = 161), UK CHIC data was used in the final dataset (as ethnicity is reported multiple times for women seen in multiple years in UK CHIC). Where there was a strong discrepancy (n = 17), such as ‘black’ versus ‘white’, ethnicity was categorised as ‘not known’ (n = 8). These records had been checked during the matching process and had sufficient matching data in other fields, including COB, to indicate that they were a genuine match.

### Data analysis

Matching and data analysis was carried out using SAS v 9.1 (SAS Institute Inc. Cary, NC, USA). HIV-positive women with a pregnancy (reported to NSHPC) whose record was found in UK CHIC (referred to as ’matched’) were compared to HIV-positive women with a pregnancy who were not found in UK CHIC (referred to as ‘non-matched’), to indicate whether women in UK CHIC with a pregnancy were representative of HIV-positive women with a pregnancy. Logistic regression was used to compare characteristics and Mann–Whitney test was used to compare median ages.

National HIV surveillance data from the Survey of Prevalent HIV Infections Diagnosed (SOPHID) were used to estimate the proportion of women seen for HIV-related clinical care in the UK included in UK CHIC. SOPHID was also used in combination with NSHPC data to estimate the completeness of matching [21].

## Results

Of the 8286 women reported to UK CHIC, 24.9% (n = 2063) had a record in the NSHPC dataset, indicating that they had ever had a pregnancy in the UK when HIV-positive. The records for these women were merged to create a ‘combined dataset’. The majority of matching records were identified using exact CD4 date or CD4 date ±30 days (Table 1).

**Table 1:** Criteria used to find records for HIV-positive women reported to NSHPC and UK CHIC

**Criteria used to find records (All pairs of records have the same DOB)**		**Records matched (n = 2063)**		
		**N**	**%**	**Cumulative%**
Exact CD4 date	CD4 ± 10 cells/mm^3^	1492	72.3	72.3
	HIV diagnosis date/site	39	1.9	74.2
CD4 date ±30 days	CD4 ± 10 cells/mm^3^	251	12.2	86.4
	HIV diagnosis date/site	59	2.9	89.2
ART drug start and stop dates	Exact dates	64	3.1	92.3
	Dates ±7 days	48	2.3	94.7
Manual	Site match	58	2.8	97.5
	Diagnosis date ±30 days	4	0.2	97.7
	Drug start or stop dates	2	0.1	97.8
	Soundex	7	0.3	98.1
	ART profile	39	1.9	100.0

### Characteristics of women in the combined dataset

Nearly three-quarters of women in the combined dataset were black-African, most were born in Africa and the majority were infected via heterosexual sex (Table 2). Less than half were HIV-diagnosed during their first reported pregnancy and 21 were diagnosed perinatally (12 of these women had a subsequent pregnancy). The majority of pregnancies resulted in a live birth (Table 2) and the median number of pregnancies was 1 (range 1, 6). There were 3035 pregnancies in total, the number increasing from 159 in 2000 to 280 in 2009. Most women (92.1%, n = 1899) attended HIV-clinical care and antenatal care at the same hospital.

**Table 2:** Characteristics of women in the combined dataset (n = 2063)

		**n**	**%**
Ethnicity	Black-African	1535	74.4
	White	243	11.8
	Black-Caribbean	104	5.0
	Other	173	8.4
	Missing	8	0.4
Region of birth*	African	1527	74.0
	European	347	16.8
	Region of the Americas	87	4.2
	Eastern Mediterranean	53	2.6
	South-East Asia	25	1.2
	Western Pacific	16	0.8
	NK	8	0.4
Probable route of infection^†^	Heterosexual sex	1798	87.2
	Injecting drug use	40	1.9
	Other	135	6.5
	NK	90	4.4
Age at start of first pregnancy (years)	Median (IQR)	30.4 (26.5-34.3)	
	Range	14-49	
HIV-diagnosis in relation to first reported pregnancy	Before pregnancy	1131	54.8
	During first pregnancy	911	44.2
	At delivery	21	1.0
Pregnancy outcome (all pregnancies, n = 3035)	Live birth	2632	86.7
	Miscarriage	178	5.9
	Termination	113	3.7
	Stillbirth	33	1.1
	Ectopic pregnancy	4	0.1
	Continuing to term	75	2.5

* WHO World Regions [31].

^†^ Using UK CHIC categories and data.

### Completeness of matching

The number of women (aged 16–49 years) in the UK CHIC dataset increased yearly; from 2036 in 1996 to 4755 in 2009, totalling 45,768 person years and representing approximately 29.5% (37,577/127,267 person years) of HIV-positive women (aged 16–49) attending HIV care in the UK in 2000–2009 [21].

In 2009, there 19,312 women (aged 16–49) seen for HIV-clinical care in the UK (according to national HIV-surveillance data) [14] and 1198 HIV-positive women with a pregnancy (1211 pregnancies) starting that year (according to the NSHPC dataset used in this study), indicating that approximately 6.2% (1198/19,312) of women seen for HIV-care in 2009 became pregnant that year. We would therefore anticipate that 279–311 women (95% confidence interval for 6.2% of 4755) in the UK CHIC dataset had a pregnancy in 2009. The combined dataset contained 275 women with a pregnancy in 2009, lower than the anticipated range.

Of the records linked using DOB which did not meet the matching criteria (4916 pairs; 3014 UK CHIC records and 3285 NSHPC records, many of which linked to multiple records with the same DOB), 137 (2.8%) pairs had ever been seen at the same site for antenatal and routine HIV care and had clinical data in UK CHIC at the time they were pregnant. Over half of these (53.3%, 73/137) had CD4 data reported to NSHPC, but only 4 were within 30 days of a CD4 date in UK CHIC and these had discrepant CD4 counts and HIV-diagnosis dates.

### Availability of pre and post-pregnancy clinical data

Half (49.6%, n = 1024) the women in the combined dataset had data in UK CHIC prior to their first reported pregnancy; these women had clinical data in UK CHIC for a median of 2.8 (IQR 1.2-5.4) years before the pregnancy. The majority of pregnancies (starting before 2009) had CD4 or viral load data in UK CHIC following the pregnancy (92.0%, 2471/2685), for a median of 3.8 (IQR 1.8-6.4) years and the median time between delivery and next viral load or CD4 assessment was 1.8 (IQR 1.1-3.5) months. The majority of pregnancies with no postnatal data in UK CHIC, resulted in a live-birth (92.5%, 198/214) and less than half (36.0%, 77/214) had data in UK CHIC before the pregnancy. As no data on departure from the UK was available it was not possible to determine whether women with no post-delivery data had left the UK. However, women with no postnatal data did not significantly differ from women with postnatal data in the proportion with a UK date of arrival (61.0% (1523/2496) compared to 60.8% (115/189), Chi-squared test p = 0.96) or the median time between UK arrival and giving birth (4.1 (IQR 2.0-7.3) compared to 3.0 (IQR 1.0-5.8) years, Mann–Whitney test p < 0.20).

### Representativeness of pregnant women in UK CHIC

Women found in both NSHPC and UK CHIC, referred to as ‘matched’ (n = 2063) differed in some ways from women in NSHPC only, referred to as ‘non-matched’ (n = 6869). A smaller proportion of matched than non-matched women had a first pregnancy where the outcome had not yet been reported (i.e. outcome was reported as ‘continuing to term’); 1.5% (n = 30) compared to 5.0% (n = 342) respectively, OR 0.28 [0.19-0.41], p < 0.001); the majority of pregnancies continuing to term started in 2009/10 (73%, 273/372). When first pregnancies with an ‘other/missing’ outcome (i.e. women who left the UK or who were lost to follow-up, 6 non-matched records and 0 matched) and pregnancies where outcome was not yet reported were excluded, the outcomes for first pregnancies were similar for matched and non-matched women (Chi-squared test p = 0.15), with 90.2% (1834/2033) compared to 88.7% (5782/6521) resulting in a live birth respectively.

Timing of HIV-diagnosis varied between matched and non-matched women; with 54.8% (n = 1131) diagnosed before their first reported pregnancy compared to 47.7% (n = 3278) respectively, OR 1.34 [1.21-1.48], p < 0.001). A somewhat higher proportion of matched than non-matched women had repeat pregnancies; 35.9% (n = 741) compared to 21.9% (n = 1502) respectively, OR 2.00 [1.80-2.23], p < 0.001).

Matched women were more likely to attend antenatal care in London than non-matched women (83.2% (n = 1717) compared to 36.8% (n = 2530) respectively, OR 8.5 [7.5-9.6], p < 0.001) and were slightly older at the start of their first pregnancy (median age: matched women 30.4 (IQR 26.5-34.3) years, non-matched women 29.6 (IQR 25.8-33.6) years, p < 0.001). Ethnicity varied somewhat - a smaller proportion of matched women were black-African compared to non-matched women (74.4% (n = 1535) compared to 78.1% (n = 5362), OR 0.82 [0.73-0.92], p < 0.001); this difference remained significant when ‘ever seen for antenatal care in London’ was included in the model (AOR 0.67 [0.58-0.76], p < 0.001). A higher proportion of matched women were black-Caribbean than non-matched women (5.0% (n = 104) compared to 3.4% (n = 230) respectively, OR 1.53 [1.21-1.94], p < 0.001), but this difference was attenuated after adjustment for antenatal care in London (AOR 1.00 [0.78-1.29], p = 0.99). The proportion of women who were white was similar among matched and non-matched women (11.8% (n = 243) and 13.4% (n = 919) respectively, OR 0.86 [0.74-1.01], p = 0.06 and AOR 1.4 [1.18-1.65] p < 0.001 after adjustment for antenatal care in London).

In the UK CHIC dataset, 84.6% (7014/8286) of women had ever attended care in London, and of those attending care in 2009, 82.1% (3906/4755) went to a London site. In the NSHPC dataset, 47.6%, (4247/8932) of women had ever had antenatal care in London. Women attending antenatal care in London differed somewhat from women attending care elsewhere, for example, they were older at the start of their first pregnancy (31.1 and 29.6 years respectively, p < 0.001), more likely to be black-African or black-Caribbean and less likely to be white than women attending care outside London (black-African: 79.8% (3390/4247) compared to 74.9% (3507/4685), OR 1.3 [1.2-1.5], p < 0.001; black-Caribbean: 5.7% (n = 240) compared to 2.0% (n = 94), OR 2.9 [2.3-3.7], p < 0.001; and white: 8.1% (n = 346) compared to 17.4% (n = 816), OR 0.42 [0.37-0.48], p < 0.001 respectively). The proportion of women diagnosed before their first pregnancy was similar for women seen in London and seen elsewhere, (48.5% (2061/4247) and 50.5% (2366/4685) respectively, Chi-squared test, p = 0.06).

## Discussion

Using deterministic decision criteria based on demographic data and clinical dates collected by NSHPC and UK CHIC we were able to determine that as a minimum estimate almost one-quarter of women who received HIV-clinical care at UK CHIC sites in 1996–2009 had a pregnancy. This method combined the use of automated matching with manual review of selected records, as has been used elsewhere [22-26] and can be repeated in future years.

As no ‘gold-standard’ was available to calculate the completeness of the matching, national HIV surveillance data of individuals attending HIV-related care, was used to estimate the expected number of women with a pregnancy in the UK CHIC dataset. The number of women with a pregnancy in our combined dataset was less than the anticipated range in 2009, indicating that there was a high but incomplete level of matching. This estimation assumes that all women in the NSHPC are reported to SOPHID, which previous linkage studies indicate is not the case [27], so the true level of matching may be higher than this estimate. A large number of records had identical DOB but were not matched as they did not meet the matching criteria. It is unlikely that many of these were genuine matches as we would expect some women to share birth dates given the number of women in both datasets, particularly as women who do not know their DOB sometimes use common proxy dates, for example where the date matches the month (1^st^ January, 2^nd^ February, etc.) [28]. Records with identical dates of birth which matched on site but no other variables (137 pairs) may have been genuine matches; however, for this dataset under-matching is preferable to creating false matches. We anticipate that with the inclusion of additional data for women with repeat pregnancies and developments in software and data collection at clinics there will be more complete matching in future years.

There are a number of limitations to the methodology, including the use of blocking to select records, in this instance DOB. This is effective at limiting the records in the matching process to those likely to be matches and is frequently used in matching large datasets [24,29,30]. However, it means that incorrect or inconsistent reporting of DOB results in a record being excluded; which may be more common among some groups than others, potentially introducing bias [28,31]. Use of demographic data for record matching, such as age, ethnicity, and COB, within any matching algorithm are likely to create some false matches. Given our study population, multiple women had the same ethnicity, COB, and age, so the additional use of clinical data was crucial for matching. However, this resulted in some selection bias, as women with more clinical data, either because they had been diagnosed prior to pregnancy or had repeat pregnancies, were more likely to be matched also indicating that the matching was somewhat incomplete. Other differences between matched and non-matched women, such as age at first pregnancy, could be attributed to the difference in the proportion attending care in London, as much of the UK CHIC data comes from London sites. The differences in ethnicity between matched and non-matched women may also be explained by differences in ethnicity between women attending care in and outside London. However, when taking this into account, black-African women were less likely to be matched than women of other ethnicities and white women were more likely to be matched.

Data discrepancies in fields common to both studies were harmonized where possible, or else categorized as ‘not known’. Discrepancies were unlikely to be a result of incorrect matching, as matched records with strong discrepancies were manually checked for additional matching variables. A woman’s antenatal data, used for completing the NSHPC reporting form, and HIV clinical data extracted for inclusion in UK CHIC, are typically stored separately, even within the same hospital, in order to maintain patient confidentiality. Reasons why these databases might be discrepant include incorrect or incomplete recording of data and inconsistent or inaccurate reporting by patients, for example where language is a problem or DOB is unknown [28].

This matching approach could be replicated in other settings, specifically large datasets which contain some or all of the same individuals and which include common clinical and demographic variables but no unique identifiers, for example, investigating the transition from adolescent to adult HIV-care by matching these separate datasets. Combining two datasets can lead to problems, as experienced here, such as discrepancies in variables available in both datasets and may introduce bias in matching records containing more clinical data. Nevertheless, the combining of datasets can provide the opportunity to study data not available from either study alone. Combining NSHPC with UK CHIC allows the study of predictors of pregnancy and changes in pregnancy incidence over time among women accessing HIV-care [32] and provides the opportunity to investigate the long-term impact of antenatal ART use on the woman’s health and future treatment responses.

## Conclusions

This matching process, used to identify HIV-positive women reported to NSHPC and UK CHIC, shows that with well considered use of demographic data and clinical dates, combined with careful manual review, it is possible to merge data from independent studies, providing useful data not available from either dataset alone.

## Abbreviations

ART: Antiretroviral therapy; COB: Country of birth; DOB: Date of birth; NSHPC: National Study of HIV in Pregnancy and Childhood; ROB: Region of birth; SOPHID: Survey of Prevalent HIV Infections Diagnosed; UK CHIC: the UK Collaborative HIV Cohort.

## Competing interests

The authors declare that they have no competing interests.

## Authors' contributions

SH developed the methodology in collaboration with LB and CT. SH carried out the statistical analysis and drafted the manuscript. CT, PT and CS advised on the analysis and interpretation. All other authors contributed to the drafting of the manuscript. All authors read and approved the final manuscript.

## Pre-publication history

The pre-publication history for this paper can be accessed here:

http://www.biomedcentral.com/1471-2288/12/110/prepub
